# Callus and etiolation induction data from explants of *Solanecio biafrae* (Olive & Hierne) C. Jeffrey cultured in the dark

**DOI:** 10.1016/j.dib.2018.07.029

**Published:** 2018-07-23

**Authors:** O.A. Bello, O. Fajimi, E.B. Esan, O.O. Obembe

**Affiliations:** a Department of Biological Sciences, College of Science and Technology, Covenant University, PMB 1023 Canaanland Ota, Ogun State, Nigeria; b Biotechnology unit, National Centre for Genetic Resources and Biotechnology. Moor Plantation, Ibadan, Oyo State, Nigeria; c Department of Basic Sciences, Babcock University, Ilishan-Remo, Ogun State, Nigeria

## Abstract

Different types of explant (leaf, nodal and petiole explant) from in vitro grown plant maintained on Murashige and Skoog (MS) medium, were cultured on MS medium supplemented with various concentrations of 2, 4-Dichlorophenoxy acetic acid(2, 4-D) (0, 0.5, 1.0, 1.5 and 2.0 mg/l) in dark condition. Data on callus formation was recorded on 10 days after culture. Number of explants forming callus, callus colour and type were recorded. The plant growth regulator-free media which served as the control induced etiolation resulting in long hypocotyls from the nodal explants.

**Specifications Table**

**Table:** 

Subject area	*Biology*
More specific subject area	*Plant Cell and Tissue culture*
Type of data	*Table and figure*
How data was acquired	*Aseptically transferred in laminar flow hood and grown in plant growth chamber*
Data format	*Raw and analyzed*
Experimental factors	*Nodal explants from potted plants were cultured on Murashige and Skoog(MS) medium to get axenic in vitro grown plantlet.*
Experimental features	*Leaf, nodal and petiole explants from in vitro grown plantlet were cultured on Murashige and Skoog(MS) medium supplemented with varied concentrations of 2,4-Dichlorophenoxyacetic acid (2,4-D) in dark condition. 10 replicates in a completely randomized design.*
Data source location	*Plant tissue culture laboratory of Covenant University, Ota. Ogun State. Nigeria.*
Data accessibility	*Data are presented in this article.*

**Value of the data**

• The data furnishes scientific body with information on callus induction of *Solanecio biafrae* using the concentrations which brings about desired morphogenic response
• This data on etiolation offers the advantages of promoting the development of axillary buds and reducing the level of fungal and viral infection at the apices.
• This data is valuable for further study on developing efficient regeneration protocol for *S. biafrae*.

## 1 Data

**Fig. 1:**
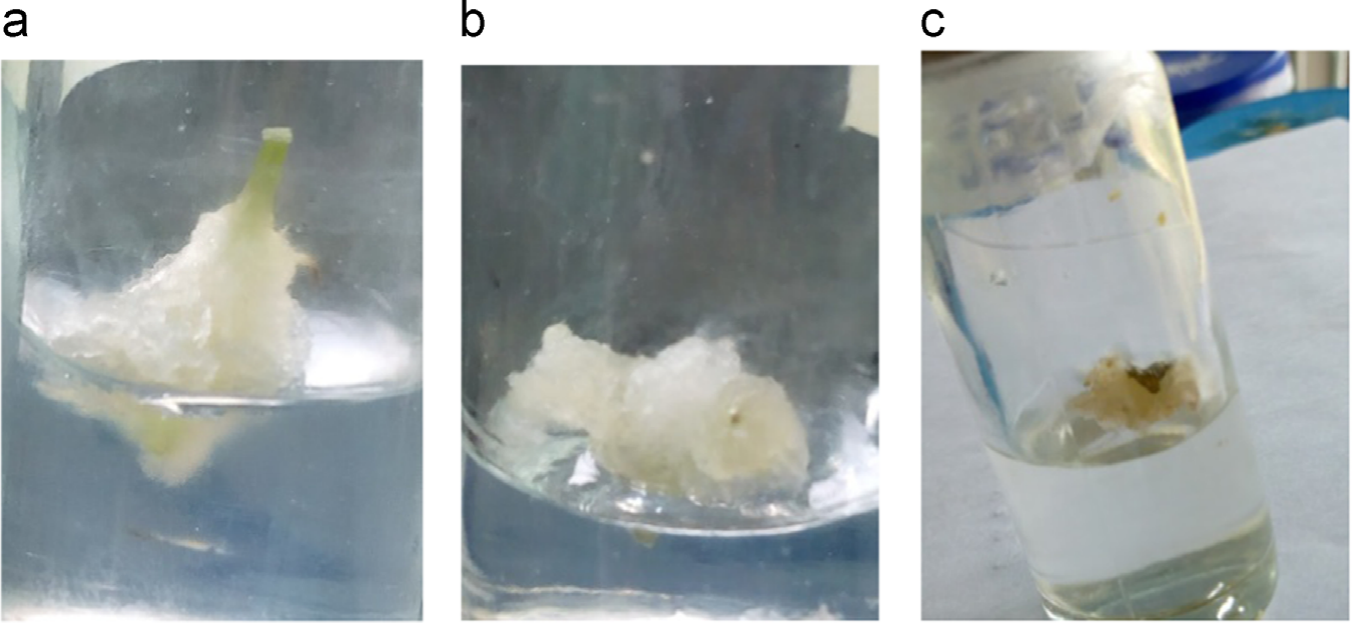
Callus formation in 2,4-Dichlorophenoxyacetic acid by (a.) nodal (b.) petiole and (c.) leaf explants.

**Fig. 2:**
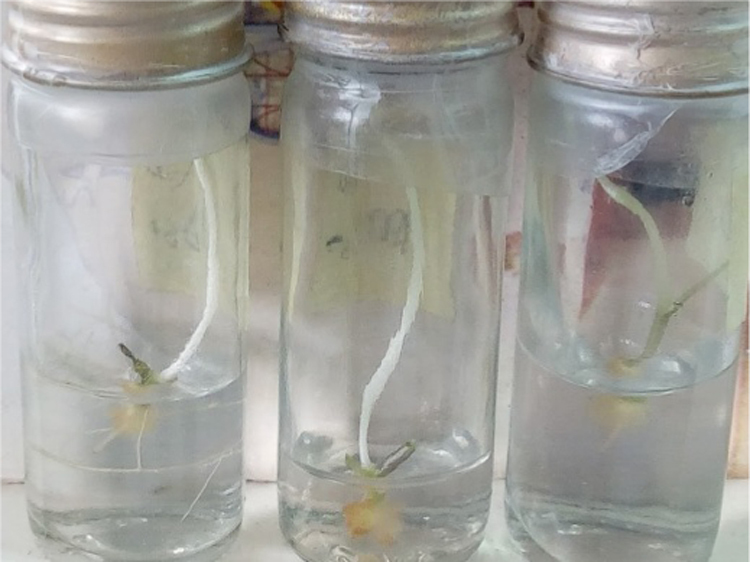
Etiolation formation in plant growth regulator-free medium grown under dark condition.

In the data, effect of 2, 4-Dichlorophenoxy acetic acid on callus formation is shown for three explant types from *Solanecio biafrae* plant grown *in vitro* under the dark condition. The age of the explant source for plant tissue culture is significant with preference to younger plants. The age barrier is therefore overcome with the use of in vitro grown plantlets (1). Fig. 1a-c represents the images of callus being formed by different explants type (nodal, petiole and leaf explants). Callus formation was observed on 10 days after culture. Meanwhile, Fig. 2 represents the image of etiolated shoot formation with an average of 4 roots formed from nodal explants cultured on MS basal medium without 2, 4-D (experimental control). Moreover, Tables 1 and 2 show the callogenic and morphogenic responses of *S*. *biafrae* explant types to the varied concentrations of 2, 4- D.

**Table 1:** Callogenic response of three explants type of *S. biafrae* on 2, 4-D in dark condition.

**Treatments**	**Response**	**Callus colour**	**Callus type**
LMD0	–	–	–
LMD1	++	Cream/Yellow	Friable
LMD2	++	Green	Friable
LMD3	++	Cream	Friable
LMD4	+++	Cream	Friable
NMD0	–	–	–
NMD1	++++	Cream	Friable
NMD2	++++	Cream	Compact
NMD3	++++	Cream	Friable
NMD4	++++	Cream	Friable
PMD0	–	–	–
PMD1	–	–	–
PMD2	++++	Cream	Friable
PMD3	++	Cream	Friable
PMD4	+	Cream	Friable

− = no response; + = 0–25%, ++ = 26–50%, 51–75%, 76–100%.

**Table 2:** Morphogenic response of three explants type of *S. biafrae* on 2, 4-D in dark condition.

**Treatments**	**Shoot response**	**Shoot number**	**Shoot length (cm)**	**Root number**	**Root length**	**Remarks**
LMD0	+	1	5.50 ± 0.50	4	0.60 ± 0.10	Etiolated shoot and root formed
LMD1	–	–	–	–	–	No shoot or root formed
LMD2	–	–	–	–	–	No shoot or root formed
LMD3	–	–	–	–	–	No shoot or root formed
LMD4	–	–	–	–	–	No shoot or root formed
NMD0	–	–	–	–	–	No shoot or root formed
NMD1	–	–	–	–	–	No shoot or root formed
NMD2	–	–	–	–	–	No shoot or root formed
NMD3	–	–	–	–	–	No shoot or root formed
NMD4	–	–	–	–	–	No shoot or root formed
PMD0	–	–	–	–	–	No shoot or root formed
PMD1	–	–	–	–	–	No shoot or root formed
PMD2	–	–	–	–	–	No shoot or root formed
PMD3	–	–	–	–	–	No shoot or root formed
PMD4	–	–	–	–	–	No shoot or root formed

## 2 Experimental design, materials and methods

### 2.1 Source of primary biological material and disinfection

Plants of *S. biafrae* were purchased from Lafenwa market in Abeokuta and cultivated via stem cutting in pots under the tree at the College of Science and Technology of Covenant University, Ota, Ogun State. Nodal segments was collected from the potted plants and disinfected under sterile conditions inside a laminar air flow cabinet. The nodal segments were surface sterilized by immersion in 70% ethanol for 5 min, and then immersed in 10% sodium hypochlorite (NaOCl) for 20 min, followed by 5% sodium hypochlorite shaken periodically for 5 min. They were then rinsed several times with sterile distilled water (SDW).

### 2.2 *In vitro* culture of *Solanecio biafrae*

All the sterilized single node explants were trimmed and cultured on Murashige and Skoog (MS) basal medium [1] supplemented with 3% sucrose. The pH was adjusted to 5.7 using NaOH or HCl before autoclaving at 121 °C for 15 min and adding 0.8% agar. All cultures were maintained at 16 hr photoperiod with 3000 lx light intensity at 25±2 °C.

### 2.3 Effect of 2,4-D on morphogenesis of *S. biafrae*

Leaf, nodal and petiole explants were excised from the *in vitro* grown stock plants maintained on MS basal medium, were cultured on MS medium supplemented with 2, 4-Dichlorophenoxy acetic acid in dark condition to study the morphogenic response of S. biafrae. The treatment with no plant growth regulator was taken as the control and evaluated. Varied concentrations of 2,4-D were added to the media before the pH was adjusted to 5.8 using NaOH or HCl, autoclaved at 121 °C for 15 min and 0.7% agar added. The treatments including control (MS basal medium only) are illustrated in Table 3. The effect of adding no plant growth regulator was taken as the control and evaluated. A total of 15 treatments were assessed for the plant. For each treatment, a minimum of 10 cultures were raised. The cultures were examined periodically and visual observation made on the morphological changes.

**Table 3:** Media treatments with different concentrations of PGR used for this study.

**Treatments**	**Explants**	**Media**
LMD0	Leaf	MS only
LMD1	Leaf	MS+0.5 mg/l 2,4-D
LMD2	Leaf	MS+1.0 mg/l 2,4-D
LMD3	Leaf	MS+1.5 mg/l 2,4-D
LMD4	Leaf	MS+2.0 mg/l 2,4-D
NMD0	Nodal	MS only
NMD1	Nodal	MS+0.5 mg/l 2,4-D
NMD2	Nodal	MS+1.0 mg/l 2,4-D
NMD3	Nodal	MS+1.5 mg/l 2,4-D
NMD4	Nodal	MS+2.0 mg/l 2,4-D
PMD0	Petiole	MS only
PMD1	Petiole	MS+0.5 mg/l 2,4-D
PMD2	Petiole	MS+1.0 mg/l 2,4-D
PMD3	Petiole	MS+1.5 mg/l 2,4-D
PMD4	Petiole	MS+2.0 mg/l 2,4-D
