# Supplementary material for: Callus and etiolation induction data from explants of Solanecio biafrae (Olive & Hierne) C. Jeffrey cultured in the dark
Source: Data Brief. 2018 Jul 23;20:113–7. doi: 10.1016/j.dib.2018.07.029 (PMC6088559; doi:10.1016/j.dib.2018.07.029)
Supplement: Supplementary file 1 — Supplementary material [file mmc1.docx]

Declarations of interest

All the authors confirm on no conflict of interest.
